# The “Smartness” of Worms and Fish

**Published:** 1882-06

**Authors:** 


### THE "SMARTNESS" OF WORMS AND FISH.

"I have made some of my most interesting studies of nature in the morning," said Seth Green. "That is the time to see the insects at their best—to see the mud wasps stinging the spiders without killing them, and packing them away where they are kept alive for weeks to be used when needed. I have seen a small green worm hanging down on a web. An ant, stationed on the limb above, pulls up the web, and, just as the worm comes within reach of his tiny claws, down drops Mr. Worm. The ant pulls up again and again and worm lets out another reef and goes down. This sort of thing continues until finally the ant grapples the worm and both go down together in a grand scramble, in which the worm manages to shake off the ant. This leaves the worm on the ground. His web is so strong that the other end is still fastened to the limb above. What does Mr. Ant do? Give it up? No, sir. I have seen him go up the trunk of that tree, crawl out onto the same limb and go to work again pulling up the same web. Then after another battle, I have known the ant to get the better of the fight and lug the worm off, to his hole, three rods away.

"Why, talk about reasoning powers! The perseverance and instinct of these little creatures are wonderful. People go out to fish. They splash around, stand up in the boat, drop their lines three feet away, and wonder because they don't catch trout. They forget that trout can see. Fish learn the tackle and fish are, as a rule, local in their habitation. There are not as many gypsies among fish as among men. Any man who will take the pains to study fish—or who will remember a tithe of what he reads about them, can catch them. They are smart, but our brains will beat them. I remember once of fishing for salmon trout for a long time and taking nothing. Finally I concluded to get down and look into the water, and so, throwing my coat over my head, I got the required shade and peered down. The salmon would sail up and look at the minnow. Then, with a quick dart, he would close his teeth round one-half the minnow and open them again like a flash. He did not attempt to eat the minnow, and half of the severed body would drop to the bottom. When it had fallen to the bed of the lake the salmon would go down leisurely and eat it. The next time when I dropped my hook and felt the quick bite of the trout I let out enough line to send the hook to the bottom, and the result was that when the salmon went down for his meal he was fooled and I had him."—*Utica Observer.*

